# Susceptibility status of *Anopheles arabiensis* (Diptera: Culicidae) commonly used as biological materials for evaluations of malaria vector control tools in Madagascar

**DOI:** 10.1186/s12936-016-1406-3

**Published:** 2016-06-30

**Authors:** Sanjiarizaha Randriamaherijaona, Haja Johnson Velonirina, Sébastien Boyer

**Affiliations:** Unité d’Entomologie Médicale, Institut Pasteur de Madagascar, Antananarivo, Madagascar; Ecole Doctorale Science de la vie et de l’environnement, Faculté des Sciences, Université d’Antananarivo, Antananarivo, Madagascar

**Keywords:** *Anopheles arabiensis*, Susceptible strain, Madagascar, Vector control, Insecticides

## Abstract

**Background:**

Madagascar is a malaria-endemic country with an increase in cases in recent years. In vector control using insecticide, a susceptible strain is necessary to evaluate insecticide efficacy, either for spraying or on nets. The susceptibility of *Anopheles arabiensis* from Antananarivo, Madagascar to two organophosphate, three pyrethroid, two carbamate, and one organochlorine insecticides was investigated. Since 2010, *An. arabiensis* strain has been maintained away from insecticide source during 110 generations with optimal insectarium conditions.

**Methods:**

Bioassay were performed on adult mosquitoes to assess the susceptibility of *An. arabiensis* to insecticide-impregnated papers (malathion 5 %, fenitrothion 1 %, deltamethrin 0.05 %, permethrin 0.75 %, alphacypermethrin 0.05 %, bendiocarb 0.1 %, propoxur 0.01 %, and DDT 4 %) following World Health Organization Pesticide Evaluation Scheme guidelines. Bioassay using Center for Disease Control bottle tests were also used to detect mortality. Molecular assay were carried out to detect the presence of knock down resistance (*kdr*) mutation using PCR techniques.

**Results:**

*Anopheles arabiensis* is fully susceptible with 100 % mortality to malathion, fenitrothion, deltamethrin, permethrin, alphacypermethrin, bendiocarb, propoxur, and DDT. No *kdr* gene was detected using PCR method.

**Conclusion:**

The strain *An. arabiensis* maintained in the insectarium of Institut Pasteur de Madagascar is a fully susceptible strain and can be used for insecticide evaluation.

## Background

*Anopheles gambiae s.s.*, *Anopheles arabiensis*, *Anopheles mascarensis*, *Anopheles funestus*, *Anopheles merus* and, recently, *Anopheles coustani* are the most important vectors of malaria in Madagascar [1–5]. Malaria vector control constitutes one of the major malaria control strategy, to target a reduction in *Anopheles* vector density and prevent parasite transmission [6] by using insecticide through indoor residual spraying (IRS) and by implementing insecticide-treated bed net (ITN) mass distribution. In sub-Saharan Africa, malaria vector control programmes continue to rely heavily on IRS and [6, 7], both of which depend on vector susceptibility to the insecticides used [8]. ITN and IRS have been proven to be effective in reducing the risk of infection with malarial parasites, clinical disease and child mortality [9–11]. In Madagascar, vector control interventions avoided over 100,000 clinical cases of malaria in 2012 and 2013 [12].

The World Health Organization (WHO) advises national programmes to evaluate insecticidal activity on nets and on treated walls [13]. Indeed, essential to the success of these vector control campaigns is the implementation of strong quality control procedures that monitor programmatic effectiveness [14–16]. Long-lasting, insecticidal-treated nets’ (LLINs) useful life may vary considerably from region to region [17, 18]. A net that is used year-round is likely to lose insecticide more rapidly due to handling and cleaning than a net that is used only seasonally [17, 19]. The efficacy of IRS may decay with time and must be re-applied frequently and it is important to know the optimal application interval in the field depending on the residual life of the insecticide [10]. Previous studies have reported that insecticide residual life depends on the substrate to which it is applied [20, 21]. Evaluation of the residual activity of insecticide applied on treated substrates becomes a necessity when aiming for long-term efficacy of an IRS implementation campaign. The World Health Organization Pesticide Evaluation Scheme (WHOPES) recommends the use of a susceptible mosquito strain, whether to evaluate LLIN bio-efficacy or to determine efficacy of the residual insecticide deposited on a wall over time. In both cases, cone bioassays are used [13, 22].

Rresults of a study is aiming to determine the susceptibility status of *An. arabiensis* which is the only laboratory strain used for assessing quality control of malaria vector control tools across Madagascar.

## Methods

### Insectarium

The insectarium is composed of a breeding room divided into a rearing-larvae box of 25 sq m and an adult-maintaining box of 15 sq m. The larvae box is sustained at a temperature of 29 °C ± 2 and adult mosquitoes are maintained at 27 °C ± 2 with a humidity of 80 %. The insectarium uses a 12:12 light:dark schedule. This is accomplished by using a simple light timer.

### Mosquitoes

The *An. arabiensis* strain has been grown at the Institut Pasteur de Madagascar since April 2010. It comes from Ambohimanambola (18°57′35.38″S; 47°35′53.91″E), southeast of Antananarivo in the Central Highlands of Madagascar. Adult mosquitoes were caught, in stables in the stage of digesting their blood meal, using manual aspirators and put into paper cups. Females were placed in cages made of netting, and their eggs were conducted into petri dishes containing cotton covered with a wet filter paper.

### *Anopheles arabiensis* rearing and colony maintaining

Eggs from wild females were reared in the insectarium. A method which allows mosquitoes to lay eggs on wet filter paper was used. The eggs were harvested every morning. Once the eggs hatched, larvae stage I were removed using a dropper and distributed in batches into white plastic trays 9 cm high × 35 cm long × 25 cm wide, containing tap water 1-cm deep. The larvae were fed with laboratory animal diet powder. To avoid water evaporation, batches were covered with a Plexiglas plate.

At emergence, mosquitoes were placed in cages 23 × 23 cm made with plastic netting. One side of the cage had an opening for allowing the arm to perform various manipulations inside the cage. During the first 20 generations, female mosquitoes were fed directly using a live rabbit. Due to restrictions on use and the difficulty of live animals in a research setting, artificial membrane methods were used: successively, pig bladder, chicken skin membrane and Parafilm M ^®^. From the 70th generations, female mosquitoes were blood-fed with healthy sheep blood by using an artificial blood-feeder (Hemotek^®^) and they received a 10 % sucrose solution.

### Insecticide susceptibility test

#### WHO bioassay tests

For each insecticide, 400 female mosquitoes 2–5 days old were exposed to diagnostic doses of various insecticides for susceptibility tests, using insecticide-impregnated papers, as described by standard WHO testing protocol [23].

Mortality resulting from tarsal contact with insecticide-treated filter papers was measured using WHO test kits [23]. The tests were carried out using malathion 5 %, fenitrothion 1 %, deltamethrin 0.05 %, permethrin 0.75 %, alphacypermethrin 0.05 %, bendiocarb 0.1 %, propoxur 0.1 %, and DDT 4 %. Insecticide-impregnated papers were obtained from the Malaysian WHO Collaborating Centre at standard concentrations for determining resistance of adult mosquitoes. Four batches of 25 unfed females were exposed to impregnated papers for 1 h. The number of knock-down mosquitoes was recorded every 10 min. Tests with untreated papers that served as control were run in parallel. At the end of the exposure period, mosquitoes were transferred into tubes with untreated white filter papers (known as holding tubes) and allowed a 24-h recovery period. All mosquitoes were provided with 10 % glucose water during the 24-h recovery period. Mortality rate was recorded after 24 h.

#### CDC bottle test

The principle of CDC bottle bioassay is to determine the time it takes an insecticide to penetrate an arthropod, traverse its intervening tissues, get to the target site, and act on that site relative to a susceptible control. Anything that prevents or delays the compound from achieving its objective of killing the arthropods contributes to resistance.

Diagnostic doses that were applied in the present study were the doses recommended by CDC [24]. For *An. gambiae s.l.*, diagnostic doses were 12.5 μg per bottle for deltamethrin and bendiocarb and 21.5, 100, 50 μg, respectively, for permethrin, DDT and malathion. The diagnostic time was 30 min except for DDT (diagnostic time = 45 min). The solutions were prepared and the bottles coated according to CDC protocol [25]. Fifteen to 25 unfed female mosquitoes aged two to five days were introduced into four 250-ml Wheaton bottles coated with insecticide and one control bottle coated with acetone only. The number of dead or alive mosquitoes was monitored at different time intervals (15, 30, 35, 40, 45, 60, 75, 90, 105, 120 min).

### PCR detection of the *kdr* mutation

One-hundred mosquitoes were used for PCR assays. Each mosquito was extracted using two or three legs following the protocol described by Cornel and Collins [26]. Leg extractions were used to genotype samples for the *kdr* allele, using a PCR diagnostic test for detection of *kdr* ‘Leu-phe’ mutations following the protocol described by Martinez-Torres [27]. Thermocycler conditions consisted of an initial denaturation step of 94 °C for 5 min, 30 cycles of 94 °C for 30 s, 50 °C for 30 s, 72 °C for 30 s, followed by a final extension of 72 °C for 5 min.

### Data interpretation

If the mortality in control batches was greater than 5 %, observed mortality rates were corrected by using Abbot’s formula [28]. Susceptibility status of *An. arabiensis* laboratory strain was determined according to the standards of WHO [24]. A mortality ranged between 98 and 100 % indicates susceptibility. An observed mortality between 90 and 97 % may indicate a resistance and resistant genes should be confirmed. If mortality is less than 90 %, the population is considered as resistant and the resistance mechanisms must be identified.

## Results

### Susceptibility status

Table 1 shows the insecticide susceptibility status of the *An. arabiensis* strain from the insectarium of the Institut Pasteur de Madagascar. *An. arabiensis* is fully susceptible when exposed to all four insecticide classes. With the two organophosphates, mortality was, respectively, 99.8 and 100 % for malathion 5 % and fenitrothion 1 %. Mortality was also high with pyrethroids, reaching 99.7 % with deltamethrin 0.05 and 100 % with both permethrin 0.75 % and alphacypermethrin 0.05 %. For carbamates, high mortality rates were observed after exposing *An. arabiensis* individuals to bendiocarb 0.1 % and propoxur 0.1 %, with mortality rates, respectively, equal to 99.5 and 99.8 %. For organochlorine, the mortality rate was 99.8 % with DDT 4 %. No dead mosquitoes were recorded when mosquitoes issued from the same strain were exposed to impregnated control paper.

**Table 1:** Susceptibility data of *Anopheles arabiensis* recorded according to both WHO and CDC methods

Classes	Insecticides	WHO test		CDC bottle test	
		N	Mortality af. 24 h (%)	N	Mortality af. diagnostic time (%)
Organophosphates (OP)	MALATHION 5 %	402	99.8	100	100
	FENITROTHION 1 %	400	100	–	–
Pyrethroids (PY)	DELTAMETHRIN 0.05 %	401	99.7	102	100
	PERMETHRIN 0.75 %	398	100	100	100
	ALPHACYPERMETHRIN 0.05 %	400	100	–	–
Carbamates (C)	BENDIOCARB 0.1 %	401	99.5	101	100
	PROPOXUR 0.01 %	400	99.8	–	–
Organochlorine (OC)	DDT 4 %	401	99.8	100	100

*Anopheles arabiensis* was fully susceptible to deltamethrin, bendiocarb, permethrin, DDT, and malathion with a mortality rate of 100 %. *An. arabiensis* laboratory strain was knock-downed after 30 min in CDC-coated bottle with deltamethrin, bendiocarb, permethrin, and malathion and after 45 min with DDT.

### Detection of *kdr* genes by PCR

**Fig. 1:**
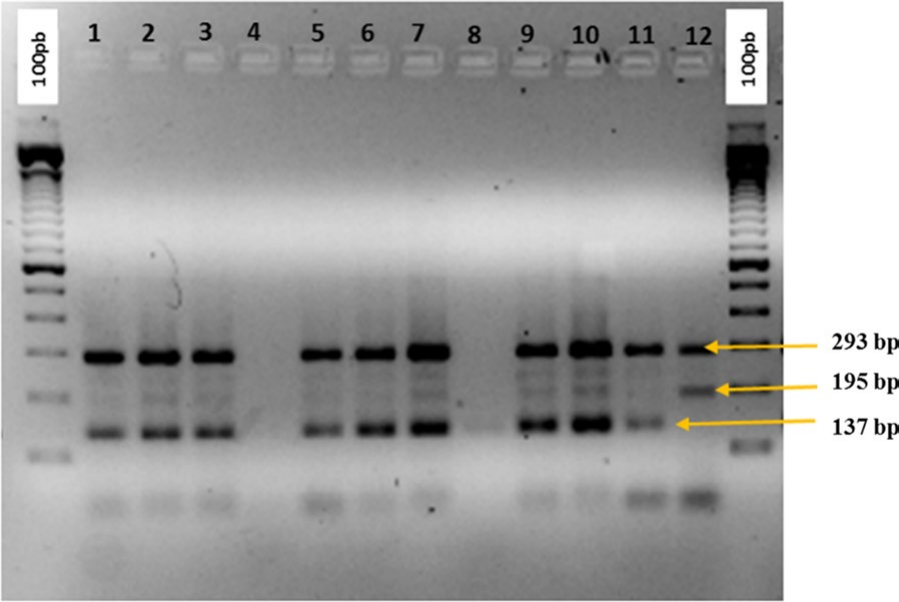
Example of PCR test diagnostic for *kdr* genotyping in *Anopheles arabiensis*. 100 pb: molecular weight ladder;* lane* 1–3; 5–7; 9–11: genomic DNA of *An. arabiensis* specimens amplified using specific primers;* lane* 4, 8: blank;* lane* 12: positive control for heterozygotes resistant genotype (R/S) with two fragments (195 bp: resistant fragment, 293 bp: susceptible fragment)

The *kdr* mutation is missing in all 100 mosquitoes tested. No band warranting resistant allele (195 bp) was detected through PCR test (Fig. 1).

## Discussion

This study describes a successful colonization of *An. arabiensis* in the laboratory. At first authors managed to establish a colony of *An. arabiensis* in Dakar (Senegal) [29]. Then, many studies focused to the improvement of *An. arabiensis* rearing, from several localities. All of these studies focused on larval development rate and wing length by studying the best larval breeding condition that would allow larval growth and survival for mass mosquito rearing [30–35]. In the current observation, the breeding productivity of *An. arabiensis* showed important difficulties to adapt in laboratory conditions. In the insectarium of Institut Pasteur de Madagascar, the percentage of hatched eggs was 40 %, which is relatively low compared to the average rate obtained with *An. arabiensis* (Dakar’s strain) in insectarium, estimated at 54.4 % [29] and increasing from generation to generation. Regarding emergence rate, results in this current study are similar to those reported by Diop et al. [29] with 95 %, allowing obtaining enough adults for the next generations.

The results of WHO bioassay test on *An. arabiensis* laboratory strain in the present study highlight the full susceptibility of this strain to insecticides. Compared to laboratory-reared *An. arabiensis* adults (KGB strain, originated from the Zambezi Valley, Zimbabwe) known to be susceptible to deltamethrin 0.05 % and bendiocarb 0.1 % [23], both populations have a mortality rate of 100 %. Using DDT 4 %, permethrin 0.75 %, the mortality rate was 100 % showing the fully susceptible status of *An. arabiensis* Institut Pasteur de Madagascar strain. The same results were obtained with the main susceptible reference strain *An. gambiae* KISUMU strain in the Republic of Cameroon [36] and in Tanzania [37].

With propoxur 0.1 % and fenitrothion 1 %, current results corroborate with results obtained with the reference strain *An. gambiae* (KISUMU strain) in Côte d’Ivoire [38] with 100 % mortality rate. No resistance was detected for the organophosphorus insecticide malathion 5 %. The mortality rate of *An. arabiensis* Institut Pasteur de Madagascar strain showed 100 % mortality just as susceptible as *An. arabiensis* Durban strain in Mozambique, with lambda-cyhalothrin 0.05 %, deltamethrin 0.05 %, permethrin 0.75 %, bendiocarb 0.01 %, propoxur 0.01 %, malathion 5 %, and DDT 4 % [39]. High mortality rates obtained with CDC bottle test corroborate the 100 % mortality rate of *An. gambiae* KISUMU strain exposed to permethrin, deltamethrin and bendiocarb [40, 41]. All in all, *An. arabiensis* Institut Pasteur de Madagascar strain shows the same susceptibility patterns as the most used susceptible *Anopheles* strains.

As preconized by WHOPES, when a compound is submitted for an evaluation, it should be tested against a susceptible reference strain, i.e., a strain which is considered to present the highest susceptibility level to the main classes of insecticides [22]. Such reference-susceptible strains exist for regionally important *Anopheles* species: *Anopheles albimanus* [42], *Anopheles darlingi* [43], *Anopheles culicifascies*, *Anopheles stephensi* [44, 45], *Anopheles quadriannulatus* [46], *Anopheles minimus* [47], *An. arabiensis* with different strains depending on the region [46, 48, 49], and *An. gambiae* Kisumu strain [50]. Considering bioassay results with *An. arabiensis* Institut Pasteur de Madagascar strain, its high susceptibility to all tested insecticides within four classes corroborate the definition of a susceptible reference strain [13, 22].

## Conclusion

This study confirmed the full susceptibility of *An. arabiensis* (Institut Pasteur de Madagascar strain). As compared to other laboratory reference strain, this strain must be considered as a reference susceptible strain, fully recommended by WHO for evaluating the quality control of malaria vector control tools.

## Abbreviations

CDC: Centers for Disease Control and Prevention
DDT: dichlorodiphenyltrichloroethane
IPM: Institut Pasteur de Madagascar
IRS: indoor residual spraying
ITN: insecticide-treated bed net
LLIN: long-lasting insecticidal net
PCR: polymerase chain reaction
WHO: World Health Organization
WHOPES: World Health Organization Evaluation Scheme

## References

[1] Chauvet G, Coz J, Gruchet H, Grjébine A, Lumaret R (1964). Contribution à l’étude biologique des vecteurs du paludisme à Madagascar: résultats de 5 années d’études (1958–1962). Med Trop (Mars)..

[2] Tsy JM, Duchemin JB, Marrama L, Rabarison P, Le Goff G, Rajaonarivelo V (2003). Distribution of the species of the *Anopheles gambiae* complex and first evidence of *Anopheles merus* as a malaria vector in Madagascar. Malar J..

[3] Le Goff G, Tsy JM, Robert V (2006). Molecular characterization of the malaria vector *Anopheles gambiae s.s*. Med Vet Entomol.

[4] Marrama L, Jambou R, Rakotoarivony I, Tsy JM, Duchemin JB, Laventure S (2004). Malaria transmission in Southern Madagascar: influence of the environment and hydro-agricultural works in sub-arid and humid regions: part 1. Entomological investigations. Acta Trop.

[5] Nepomichene TN, Tata E, Boyer S (2015). Malaria case in Madagascar, probable implication of a new vector *Anopheles coustani*. Malar J..

[6] World Health Organization (2014). World malaria report 2013.

[7] Mnzava AP, Knox TB, Temu EA, Trett A, Fornadel C, Hemingway J (2015). Implementation of the global plan for insecticide resistance management in malaria vectors: progress, challenges and the way forward. Malar J..

[8] Kleinschmidt I, Schwabe C, Shiva M, Segura JL, Sima V, Mabunda SJA (2009). Combining indoor residual spraying and insecticide-treated net interventions. Am J Trop Med Hyg.

[9] Lengeler C (2004). Insecticide-treated bed nets and curtains for preventing malaria. Cochrane Database Syst Rev..

[10] Pluess B, Tanser FC, Lengeler C, Sharp BL (2010). Indoor residual spraying for preventing malaria. Cochrane Database Syst Rev..

[11] Sharp BL, Kleinshmidt I, Streat E, Maharaj R, Barnes KI, Durrheim DN (2007). Seven years of regional malaria control collaboration-Mozambique, South Africa, and Swaziland. Am J Trop Med Hyg.

[12] Kesteman T, Randrianarivelojosia M, Raharimanga V, Randrianasolo L, Piola P, Rogier C (2016). Effectiveness of malaria control interventions in Madagascar: a nationwide case–control survey. Malar J..

[13] WHO (2013). Guidelines for laboratory and field-testing of long-lasting insecticidal nets.

[14] Green MD, Atieli F, Akogbeto M (2009). Rapid colorimetric field test to determine levels of deltamethrin on PermaNet^®^ surfaces: association with mosquito bioactivity. Trop Med Int Health..

[15] Jenkins DW, Hensens A, Lloyd J, Payne M, Cizmarik P, Hamel S (2013). Development and validation of a ‘universal’ HPLC method for pyrethroid quantification in long-lasting insecticidal mosquito nets for malaria control and prevention. Trop Med Int Health..

[16] Russell TL, Morgan JC, Ismail H, Kaur H, Eggelte T, Oladepo F (2014). Evaluating the feasibility of using insecticide quantification kits (IQK) for estimating cyanopyrethroid levels for indoor residual spraying in Vanuatu. Malar J..

[17] Allan R, O’Reilly L, Gilbos V, Kilian A (2012). An observational study of material durability of three World Health Organization-recommended long-lasting Insecticidal nets in eastern Chad. Am J Trop Med Hyg.

[18] Mattern C, Pourette D, Raboanary E, Kesteman T, Piola P, Randrianarivelojosia M (2016). “Tazomoka is not a problem”. Local perspectives on malaria, fever case management and bed net use in Madagascar. PLoS ONE.

[19] Morgan J, Abilio AP, Pondja A, Marrenjo D, Luciano J, Fernandes G (2015). Physical durability of two types of long-lasting insecticidal nets (LLINs) 3 years after a mass llin distribution campaign in Mozambique, 2008–2011. Am J Trop Med Hyg.

[20] Ansari MA, Razdan RK (2004). Impact of residual spraying of bendiocarb against the malaria vector *Anopheles culicifacies* in selected villages of the Ghaziabad District, Uttar Pradesh. India. J Am Mosq Control Assoc..

[21] Etang J, Nwane P, Mbida JA, Piameu M, Manga B, Souop D (2011). Variations of insecticide residual bio-efficacy on different types of walls: results from a community-based trial in south Cameroon. Malar J.

[22] WHO (2006). Guidelines for testing mosquito adulticides for indoor residual spraying and treatment of mosquito nets.

[23] WHO (1998). Test procedures for inseciticde resistance monitoring in malaria vectors, bio-efficacy and persistance of insecticide in treated surfaces.

[24] WHO (2013). Test procedures for insecticide resistance monitoring in malaria vector mosquitoes.

[25] CDC (2010). Guideline for evaluating insecticide resistance in vectors using the CDC bottle bioassay.

[26] Cornel AJ, Collins FH, Clapp JP (1996). PCR of the ribosomal DNA intergenic spacer regions as a method for identifying mosquitoes in the *Anopheles gambiae* complex. Methods in molecular biology.

[27] Martinez-Torres D, Chandre F, Williamson MS, Darriet F, Berge JB, Devonshire AL (1998). Molecular characterization of pyrethroid knockdown resistance (*kdr*) in the major malaria vector *Anopheles gambiae s.s*. Insect Mol Biol.

[28] Abbott WS (1987). A method of computing the effectiveness of an insecticide. J Am Mosq Control Assoc..

[29] Diop A, Faye O, Molez JF (1998). Colonization in insectarium of a strain of *Anopheles arabiensis* (Diptera: Culicidae). Bull Soc Pathol Exot.

[30] Balestrino F, Soliban SM, Gilles J, Oliva C, Benedict MQ (2010). Ovipositional behavior in the context of mass rearing of *Anopheles arabiensis*. J Am Mosq Control Assoc..

[31] Gilles JRL, Lees RS, Soliban SM, Benedict MQ (2011). Density-dependent effects in experimental larval populations of *Anopheles arabiensis* (Diptera: Culicidae) can be negative, neutral, or overcompensatory depending on density and diet levels. J Med Entomol.

[32] Kirby MJ, Lindsay SW (2009). Effect of temperature and inter-specific competition on the development and survival of *Anopheles gambiae* sensu stricto and *An. arabiensis* larvae. Acta Trop.

[33] Damiens D, Benedict MQ, Wille M, Gilles JRL (2012). An inexpensive and effective larval diet for *Anopheles arabiensis* (Diptera: Culicidae): eat like a horse, a bird, or a fish?. J Med Entomol.

[34] Khan I, Vreysen M (2011). Comparing Efficacy of mixed larval diets on the developmental attributes of *Anopheles arabiensis* Patton. Pak J Zool..

[35] Mamai W, Lees RS, Maiga H, Gilles JR (2016). Reusing larval rearing water and its effect on development and quality of *Anopheles arabiensis* mosquitoes. Malar J..

[36] Etang J, Manga L, Chandre F, Guillet P, Fondjo E, Mimpfoundi R (2003). Insecticide susceptibility status of *Anopheles gambiae s.l.* (Diptera: Culicidae) in the Republic of Cameroon. J Med Entomol.

[37] Kabula B, Tungu P, Matowo J, Kitau J, Mweya C, Emidi B (2012). Susceptibility status of malaria vectors to insecticides commonly used for malaria control in Tanzania. Trop Med Int Health..

[38] Alou LPA, Koffi AA, Adja MA, Tia E, Kouassi PK, Kone M (2010). Research distribution of ace-1R and resistance to carbamates and organophosphates in *Anopheles gambiae**s.s.* populations from Côte d’Ivoire. Malar J..

[39] Casimiro S, Coleman M, Hemingway J, Sharp B (2006). Insecticide resistance in *Anopheles arabiensis* and *Anopheles gambiae* from Mozambique. J Med Entomol.

[40] Aïzoun N, Aïkpon R, Gnanguenon V, Oussou O, Agossa F, Padonou G (2013). Status of organophosphate and carbamate resistance in *Anopheles gambiae* sensu lato from the south and north Benin West Africa. Parasit Vectors..

[41] Aïzoun N, Azondekon R, Aïkpon R, Gnanguenon V, Osse R, Asidi A (2014). Study of the efficacy of a Wheaton coated bottle with permethrin and deltamethrin in laboratory conditions and a WHO impregnated paper with bendiocarb in field conditions. Asian Pac J Trop Biomed..

[42] Jaramillo GI, Robledo PC, Mina NJ, Muñoz JA, Ocampo CB (2011). Comparison of the efficacy of long-lasting insecticidal nets PermaNet^®^ 2.0 and Olyset^®^ against *Anopheles albimanus* under laboratory conditions. Mem Inst Oswaldo Cruz.

[43] Hiwat H, Mitro S, Samjhawan A, Sardjoe P, Soekhoe T, Takken W (2012). Collapse of *Anopheles darlingi* populations in Suriname after introduction of insecticide-treated nets (ITNs); malaria down to near elimination level. Am J Trop Med Hyg.

[44] Rafinejad J, Vatandoost H, Nikpoor F, Abai MR, Shaeghi M, Duchen S (2008). Effect of washing on the bio-efficacy of insecticide-treated nets (ITNs) and long-lasting insecticidal nets (LLINs) against main malaria vector *Anopheles stephensi* by three bioassay methods. J Vector Borne Dis..

[45] Sreehari U, Raghavendra K, Rizvi MMA, Dash AP (2009). Wash resistance and efficacy of three long-lasting insecticidal nets assessed from bioassays on *Anopheles culicifacies* and *Anopheles stephensi*. Trop Med Int Health..

[46] Hargreaves K, Hunt RH, Brooke BD, Mthembu J, Weeto MM, Awolola TS (2003). *Anopheles arabiensis* and *An. quadriannulatus* resistance to DDT in South Africa. Med Vet Entomol..

[47] Prakash A, Bhattacharyya DR, Mohapatra PK, Gogoi P, Sarma DK, Bhattacharjee K (2009). Evaluation of Permanet^®^ 2.0 mosquito bednets againsts mosquitoes, including *Anopheles minimus s.l.* in India. Southeast Asian J Trop Med Public Health.

[48] Abdalla H, Wilding CS, Nardini L, Pignatelli P, Koekemoer LL, Ranson H (2014). Insecticide resistance in *Anopheles arabiensis* in Sudan: temporal trends and underlying mechanisms. Parasit Vectors..

[49] Nardini L, Christian RN, Coetzer N, Ranson H, Coetzee M, Koekemoer LL (2012). Detoxification enzymes associated with insecticide resistance in laboratory strains of *Anopheles arabiensis* of different geographic origin. Parasit Vectors..

[50] Chandre F, Darrier F, Manga L, Akogbeto M, Faye O, Mouchet J (1999). Status of pyrethroid resistance in *Anopheles gambiae* sensu *lato*. Bull World Health Organ.

